# The Influence of AI on Critical Thinking and Creativity in L2 Learning Contexts: A Social Cognitive Perspective

**DOI:** 10.3390/jintelligence14050078

**Published:** 2026-05-02

**Authors:** Yilong Yang, Shuyi Zhang, Yadan Li

**Affiliations:** 1 Research Center for Linguistics and Applied Linguistics, Xi’an International Studies University, Xi’an 710100, China; 2 School of English Studies, Xi’an International Studies University, Xi’an 710100, China; zhangshuyi0815@163.com; 3 MOE Key Laboratory of Modern Teaching Technology, Shaanxi Normal University, Xi’an 710061, China; liyadan@snnu.edu.cn

**Keywords:** AI self-efficacy, AI literacy, critical thinking, creativity, second language learning

## Abstract

The expanding role of artificial intelligence (AI) in education raises important questions about how AI-supported learning may foster higher-order thinking and creative talent development. Guided by social cognitive theory, the current research examined how AI self-efficacy predicts creativity among second language (L2) learners through the mediating roles of AI literacy and critical thinking disposition. Two substudies were conducted. Study 1 (*N* = 72) tested a simple mediation model and demonstrated that AI self-efficacy positively predicted creativity both directly and indirectly through AI literacy. Study 2 (*N* = 135) extended these findings by incorporating critical thinking disposition and by using another measure of creativity. Results showed that AI self-efficacy positively predicted creativity, and this relationship was mediated independently by AI literacy and critical thinking disposition, as well as sequentially through both factors. The current study provides empirical evidence for pathways linking AI self-efficacy, AI literacy, critical thinking disposition, and creativity in AI-supported L2 learning. It highlights the importance of reflective and critical use of AI tools in language education.

## 1. Introduction

The growing integration of artificial intelligence (AI) into language education has expanded opportunities for developing linguistic proficiency and has also prompted increasing discussion of higher-order thinking skills, particularly critical thinking (Liang & Wu, 2024). However, the cognitive mechanisms through which AI use may support creativity remain relatively underexplored in AI-supported L2 learning contexts.

Creativity involves generating original and meaningful ideas, while critical thinking enables the evaluation and refinement of those ideas (Lau, 2015; Runco & Jaeger, 2012). Previous studies suggest that these abilities are interdependent but distinct, and should be developed in parallel in educational contexts (Park et al., 2023). In AI-supported learning environments, learners’ AI self-efficacy (i.e., their confidence in using AI tools) may shape how they engage with AI resources, while AI literacy (i.e., the ability to understand and use AI) may support reflective engagement and creative thinking (Agaoglu et al., 2025; Y.-Y. Wang & Chuang, 2024).

However, it remains unclear whether and how AI self-efficacy, AI literacy, critical thinking, and creativity are associated. To address this gap, the present study investigates how AI self-efficacy predicts creativity among L2 learners and tests whether AI literacy and critical thinking disposition mediate this effect. By examining these pathways, the current study contributes to understanding how AI is linked to creative talent development in language learning within a social cognitive framework.

## 2. Literature Review

### 2.1. AI Self-Efficacy and Creativity

Self-efficacy refers to individuals’ beliefs in their capability to organize and execute actions required to achieve desired outcomes (Bandura, 1997). In technology-enhanced learning contexts, AI self-efficacy describes learners’ confidence in using AI tools for learning tasks and problem-solving (Y.-Y. Wang & Chuang, 2024). Individuals with higher AI self-efficacy are more likely to use AI tools, persist through challenges, and integrate feedback from AI systems into their study. By contrast, low self-efficacy results in avoidance of AI use, shallow interaction with AI responses, or overreliance on AI outputs.

Creativity is defined as the ability to produce original and appropriate ideas (Runco & Jaeger, 2012). It benefits from exploratory, iterative, and flexible thinking processes. Research on bilingualism has shown a positive association between multilingual experience and creativity (Acar et al., 2024). In L2 learning, this association may be related to learners’ confidence and their willingness to take risks when expressing ideas. Because AI-supported learning environments often involve uncertainty, decision-making, and inference, AI self-efficacy may shape learners’ engagement in such processes.

Creativity research indicates that confidence plays a central role in creative performance (Haase et al., 2018). Creative self-efficacy, for example, has been shown to predict creative performance in professional settings (Tierney & Farmer, 2002). Although AI self-efficacy is different, both constructs, in theory, involve efficacy beliefs that are linked to motivation and strategy use. Individuals who feel capable of using AI tools are more inclined to experiment with multiple representations, draw on diverse forms of input, and engage in iterative refinement. These are commonly regarded as characteristic of creative cognition.

However, direct empirical evidence on the association between AI self-efficacy and creativity remains limited in AI-supported L2 learning contexts. Studies exploring the roles of AI in creativity often focus on tool effectiveness or task outcomes rather than the psychological factors shaping individuals’ engagement. Recent meta-analysis and review have shown robust but variable associations between efficacy beliefs and creativity outcomes, while they also highlight possible reciprocal relationships and contextual boundary conditions (e.g., Haase et al., 2018; Herianto et al., 2024; Oh & Pyo, 2023; Tao et al., 2025; Yodchai et al., 2022). Therefore, understanding how and why AI self-efficacy predicts creativity requires examining intermediate cognitive pathways, particularly those involving AI literacy and evaluative reasoning (i.e., critical thinking disposition).

### 2.2. AI Self-Efficacy, AI Literacy, and Creativity

AI literacy refers to the knowledge, skills, and dispositions required to understand, apply, evaluate, and communicate using AI technologies (Long & Magerko, 2020; Ng et al., 2021). Current frameworks highlight four components: (1) conceptual awareness of how AI operates, (2) practical ability to use AI tools strategically, (3) critical evaluation of AI outputs, and (4) ethical and reflective judgment regarding AI’s role in learning and society (Almatrafi et al., 2024).

In educational contexts, students with higher AI literacy are better prepared to use AI tools not as authoritative sources of answers but as partners in meaning-making and inquiry (Folmeg et al., 2024). For instance, literate AI learners tend to revise prompts intentionally, compare multiple outputs, integrate external sources, and adapt AI output to task requirements. Such behaviors require both cognitive control and flexibility, and are relevant to creativity. This pattern suggests a potential association between AI literacy and creativity.

The previous studies provide indirect support for this association. Media and information literacy training enhances students’ ability to detect misinformation and engage in reasoning-based evaluation (Al Zou’bi, 2022). Digital literacy has also been shown to strengthen engagement, problem-solving, and confidence in technology-mediated learning environments (Getenet et al., 2024; Naveed et al., 2023). In specific AI-supported educational contexts, AI competencies and digital literacy may facilitate more active and creative engagement with tasks rather than the passive use (Agaoglu et al., 2025).

Social cognitive theory provides a theoretical rationale linking AI self-efficacy to AI literacy. Individuals with strong AI self-efficacy are more likely to experiment with AI tools, engage in trial-and-error learning, and sustain participation when challenges arise. These behaviors may create opportunities to develop literacy competencies through experience. Therefore, AI self-efficacy may be indirectly related to creativity through its association with AI literacy. In other words, learners with higher AI self-efficacy may engage more with AI tools (i.e., higher AI literacy), which is in turn associated with the flexible and adaptive thinking processes involved in creativity.

### 2.3. AI Self-Efficacy, Critical Thinking Disposition, and Creativity

Critical thinking disposition is defined as a habitual tendency toward purposeful and self-regulated judgment involving analysis, inference, evaluation, and decision-making based on evidence and reasoning (Facione, 1990). In L2 learning, critical thinking disposition is essential for constructing arguments, evaluating information, and forming reasoned interpretations (McKinley, 2013). It also serves as a regulatory mechanism in the creative process. While creativity generates ideas, critical thinking disposition refines them to ensure coherence, relevance, and feasibility (Jones, 2015).

In AI-assisted learning contexts, critical thinking disposition plays a particularly important role because the effects of AI use on higher-order thinking may vary across learning conditions (Deng et al., 2025). Learners must therefore evaluate when, how, and why to adopt, modify, or reject AI suggestions. Such evaluative engagement is not automatic. It depends on learners’ willingness to question surface-level fluency and to prioritize evidence-based judgment.

Research indicates that self-efficacy is associated with critical thinking dispositions. Academic and technological self-efficacy influence cognitive engagement, metacognitive monitoring, and willingness to question assumptions, all of which contribute to critical thinking disposition (Chang et al., 2022; Vachova et al., 2023). Extending this logic to AI-supported L2 learning, learners with higher AI self-efficacy may be more inclined to take an active and self-regulatory stance during human–AI interaction. Specifically, such learners are more likely to initiate evaluative behaviors, such as refining prompts, requesting clarification, comparing alternative outputs, and integrating AI suggestions rather than accepting them directly. 

Studies have also indicated positive associations between critical thinking disposition and creativity. Both capacities share underlying cognitive processes such as flexibility, metacognitive awareness, and reflective reasoning (Dwyer et al., 2025; Shaber et al., 2025). However, they are also different in their functions. Creativity facilitates the generation of ideas, while critical thinking disposition supports their evaluation and refinement (Park et al., 2023). This complementary relationship suggests that critical thinking disposition may operate as an intermediate mechanism linking AI self-efficacy to creativity. Learners with higher AI self-efficacy may therefore be more likely to engage in deliberate evaluation of AI output. This process reflects stronger critical thinking disposition, which may contribute to creative ideas that are both original and appropriate. At the same time, reciprocal relationships are theoretically possible.

### 2.4. The Present Study

The preceding review indicates that AI self-efficacy may be an important factor shaping how language learners engage with AI-supported learning environments. Learners who feel confident in their ability to use AI tools are more likely to explore available functions, persist when difficulties arise, and reflect on AI outputs rather than using them directly. However, the role of AI self-efficacy in creativity remains insufficiently explored in L2 learning. In particular, it is unclear whether AI self-efficacy is directly associated with creativity or whether this association is linked to AI literacy and critical thinking disposition.

Grounded in social cognitive theory, the present study conceptualizes AI self-efficacy as a motivational belief that may shape language learners’ engagement when they use AI. From this perspective, AI self-efficacy is also expected to be linked to creativity indirectly through learners’ AI competencies (i.e., AI literacy) and evaluative tendencies (i.e., critical thinking disposition). AI literacy is conceptualized as abilities reflecting language learners’ knowledge, skills, and reflective judgment in using AI tools, whereas critical thinking disposition refers to learners’ habitual inclination to engage in reflective evaluation. Together, these constructs provide possible pathways through which AI self-efficacy may predict creativity in AI-supported L2 learning.

To examine these relationships, two substudies were conducted. Study 1 provides an initial test of whether AI literacy mediates the association between AI self-efficacy and creativity, which was tested by a divergent thinking task. Study 2 extends this model by incorporating critical thinking disposition as an additional mediator and by measuring creativity as everyday ideational behavior. This design allows for the examination of both independent and sequential mediation pathways, as well as for testing whether the proposed associations are consistent across different measures of creativity. Based on the theoretical rationale outlined above, the present study tested the following hypotheses:

**H1.**  *AI literacy mediates the relationship between AI self-efficacy and creativity.*

**H2.**  *Critical thinking disposition mediates the relationship between AI self-efficacy and creativity.*

**H3.**  *AI literacy and critical thinking disposition serially mediate the relationship between AI self-efficacy and creativity.*

## 3. Study 1

### 3.1. Participants

Study 1 included 72 participants (*M_age_* = 19.310 ± 0.973 years, age range: 18–22 years), who were college students learning English in China (Table 1). All participants were native Mandarin Chinese speakers and had been learning English as their second language. The average age of onset for learning English was 8.236 ± 1.756 years, and participants self-reported their English proficiency with an average score of 4.125 ± 0.580 (rated on a 7-point scale). In recruitment and briefing, participants frequently mentioned using mainstream Chinese AI platforms in their study, such as Doubao, DeepSeek, and Qwen. Accordingly, the “AI tools” in the study mainly refer to these widely available chatbot services in China. All participants indicated no history of substance abuse, addiction, or mental illness. None reported prior creativity training.

### 3.2. Instruments

#### 3.2.1. The AI Self-Efficacy Scale

The Artificial Intelligence Self-Efficacy Scale (AISES; Y.-Y. Wang & Chuang, 2024) was utilized to assess participants’ perceived self-efficacy for using AI technologies. The instrument has 22 items with four dimensions (i.e., assistance, anthropomorphic interaction, comfort with AI, and technological skills). The AISES was originally developed and validated as a domain-general measure of individuals’ confidence in using AI technologies (Y.-Y. Wang & Chuang, 2024). Although it was not designed exclusively for language learning, recent studies in AI-assisted L2 contexts have applied this scale with Chinese university EFL learners and reported satisfactory reliability and theoretically consistent associations with learning outcomes (Chen et al., 2024; L. Zhou et al., 2025). In the present study, the AISES was translated from English into Chinese following a rigorous translation and review procedure involving bilingual translation, iterative revision, and expert-panel discussion to ensure semantic equivalence and contextual appropriateness for Chinese university L2 learners. In Study 1 (*N* = 72), the Chinese version of the AISES demonstrated adequate internal consistency (Cronbach’s *α* = 0.84).

#### 3.2.2. The AI Literacy Scale

The AI Literacy Scale (AILS; B. Wang et al., 2023) was used to assess participants’ general competence with AI. The finalized instrument has 12 items rated on a 7-point Likert scale. It has four constructs, namely awareness, usage, evaluation, and ethics. The scale was translated from English into Chinese by the authors. The translation was carefully reviewed and revised to ensure clarity and cultural appropriateness for the Chinese population. In the current research, the internal consistency reliability of the AILS was found to be adequate (Cronbach’s *α* = 0.75).

#### 3.2.3. The Alternative Uses Task

The Alternative Uses Task (AUT; Guilford, 1967) is a widely used objective test for creativity. In the task, participants were asked to be creative and list as many uses as possible for an umbrella, a brick, and a spoon. Each participant had five minutes for each object. AUT responses were scored for originality by two raters with research experience in creativity. Prior to formal scoring, the raters conducted a brief calibration procedure involving joint and independent scoring of a small subset of responses to establish consistent scoring criteria. Creativity was operationalized using an average originality index, calculated as the total originality score across all valid uses divided by the number of valid uses generated by each participant. Final AUT scores were obtained by averaging the two raters’ evaluations. Inter-rater reliability was satisfactory (Cronbach’s *α* = 0.81).

### 3.3. Data Analysis

SPSS (v25.0) was used for descriptive statistics and correlation analyses. Hypothesis testing employed the PROCESS macro (v3.2; Model 4) with 5000 bootstrap resamples to estimate indirect effects and 95% confidence intervals (CIs). We followed established procedures for mediation analysis (Hayes, 2022; Preacher & Hayes, 2004).

### 3.4. Results

#### 3.4.1. Descriptive Statistics and Correlation Analysis

Participants’ performance in AI self-efficacy (101.653 ± 12.907), AI literacy (65.125 ± 7.403), and creativity (5.770 ± 1.273) was evaluated (Table 2). Pearson correlations indicated positive associations among the three constructs, i.e., AI self-efficacy with AI literacy (*r* = 0.371, *p* < 0.01) and with creativity (*r* = 0.400, *p* < 0.001), and AI literacy with creativity (*r* = 0.367, *p* < 0.01).

#### 3.4.2. Mediation Analysis

**Figure 1:**
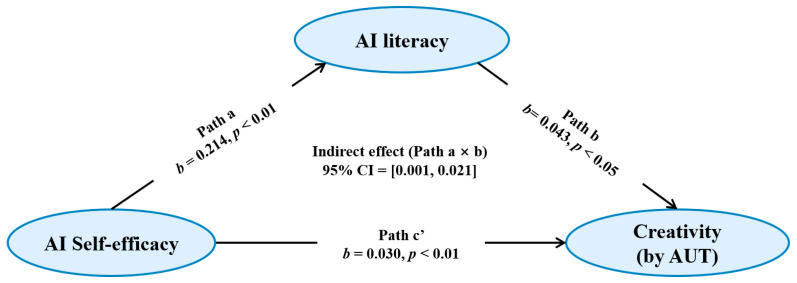
The mediation role of AI literacy in the correlation between AI self-efficacy and creativity (H1).

To test Hypothesis 1 (H1), a simple mediation analysis was performed (Figure 1). The results indicated that AI self-efficacy positively predicted AI literacy (*b* = 0.214, *SE* = 0.064, *β* = 0.371, *p* < 0.01). This model explained 13.8% of the variance in AI literacy (*R*^2^ = 0.138, *f*^2^ = 0.160). The full model, including both AI self-efficacy and AI literacy, explained 21.6% of the variance in creativity (*R*^2^ = 0.216, *f*^2^ = 0.276). Within this model, AI self-efficacy positively predicted creativity (*b* = 0.030, *SE* = 0.011, *β* = 0.306, *p* < 0.01). AI literacy positively predicted creativity (*b* = 0.043, *SE* = 0.020, *β* = 0.253, *p* < 0.05). Additionally, the bootstrapped indirect effect of AI self-efficacy on creativity via AI literacy was significant (*b* = 0.009, *SE* = 0.005, 95% CI = [0.001, 0.021]; completely standardized indirect effect = 0.094, SE = 0.046, 95% CI [0.008, 0.187]). These results are consistent with a partial indirect effect of AI self-efficacy on creativity through AI literacy.

#### 3.4.3. Post Hoc Sensitivity (Power) Analysis

We conducted a post hoc sensitivity (power) analysis in G*Power (v3.1.9.7) for the multiple-regression equation(s) underlying the PROCESS mediation step(s), focusing on the omnibus *F* test for each regression equation. Using an F-test framework (linear multiple regression, α = 0.05, Study 1 sample size *N* = 72, number of tested predictors = 1, and total number of predictors = 3), the achieved power was 0.999, with a critical *F* of 3.982. It indicates high sensitivity for detecting the observed regression-level effects in the Study 1 equation(s).

### 3.5. Validation and Robustness Checks

Because AI self-efficacy and AI literacy were evaluated via self-report in Study 1, a series of diagnostic and robustness checks was conducted.

First, the common-method variance was examined using Harman’s single-factor test across all self-report items. The first unrotated factor accounted for 26.66% of the total variance, which is below the 50% threshold. This finding suggests that common-method bias is unlikely to be a dominant concern in the present data.

Second, an assessment was conducted in order to ascertain the presence of multicollinearity in the regression equations of the mediation model. This assessment revealed that the tolerance values exceeded 0.20, while the VIF values remained below 5.00. These results indicate that multicollinearity was not a significant concern.

Third, the mediation model was re-estimated whilst controlling for available L2 background covariates (i.e., age of onset of English learning and self-rated English proficiency). The total effect of AI self-efficacy on creativity remained significant after controlling for these covariates (*b* = 0.037, *SE* = 0.011, *p* < 0.001).

### 3.6. Interim Discussion

Study 1 provided initial evidence of positive associations among AI self-efficacy, AI literacy, and creativity in L2 learners. It further indicated a mediating effect of AI literacy on the link between AI self-efficacy and creativity, providing preliminary support for the first hypothesis (H1). Rather than implying a causal relationship, these findings suggest that L2 learners with higher AI self-efficacy tend to demonstrate higher AI literacy, which is associated with stronger creativity (i.e., divergent thinking) performance. Building on theoretical and empirical work that connects AI competencies with creativity, it is necessary to examine additional cognitive mechanisms. In particular, we ask whether critical thinking disposition functions as a further mediator of the AI self-efficacy and creativity relationship. Study 2 was therefore designed to investigate this question and to test the second (H2) and third hypotheses (H3).

## 4. Study 2

### 4.1. Participants

Study 2 included 135 participants (*M_age_* = 19.470 ± 1.164 years, age range: 17–23 years), who were college students learning English in China (Table 3). All participants were native Mandarin Chinese speakers and had been learning English as their second language. The average age of onset for learning English was 8.100 ± 1.912 years, and participants self-reported their English proficiency with an average score of 4.160 ± 0.660 (rated on a 7-point scale). In recruitment and briefing, participants mentioned using mainstream Chinese AI platforms in their study, such as Doubao, DeepSeek, and Qwen. The “AI tools” in the study mainly refer to these widely available chatbot services in China. All participants indicated no history of substance abuse, addiction, or mental illness. None reported prior creativity training.

### 4.2. Instruments

#### 4.2.1. The AI Self-Efficacy Scale

In Study 2, the same Chinese version of the AISES (Y.-Y. Wang & Chuang, 2024) was administered. Internal consistency reliability in the larger sample (*N* = 135) of Study 2 remained adequate (Cronbach’s *α* = 0.84). To further examine the scale’s structural validity in the current data, an exploratory factor analysis (EFA) was conducted using principal axis factoring with oblique rotation. Sampling adequacy was satisfactory (KMO = 0.832, Bartlett’s *χ*^2^ (231) = 1973.328, *p* < 0.001). Consistent with the original four-factor conceptualization, four factors were retained, accounting for 60.33% of the extracted common variance.

#### 4.2.2. The AI Literacy Scale

In Study 2, the same Chinese version of AILS (B. Wang et al., 2023) was used to assess participants’ general competence with AI. Internal consistency reliability in the larger sample (*N* = 135) of Study 2 remained adequate (Cronbach’s *α* = 0.75). To examine the latent structure of the Chinese version of the AILS, we conducted an exploratory factor analysis (EFA) in the larger Study 2 sample (*N* = 135) using principal axis factoring (PAF) with no rotation. The data were suitable for factor analysis (KMO = 0.801, Bartlett’s test of sphericity *χ*^2^ (66) = 535.012, *p* < 0.001). Using a single composite score, we tested a one-factor model, which revealed a dominant general factor explaining 30.41% of the variance. 

#### 4.2.3. The California Critical Thinking Disposition Inventory (Chinese Version)

Critical thinking disposition was assessed with the California Critical Thinking Disposition Inventory (Chinese Version) (Peng et al., 2004). This adapted Chinese version (CTDI-CV) preserves seven dispositions, i.e., truth seeking, open-mindedness, analyticity, systematicity, critical-thinking self-confidence, inquisitiveness, and cognitive maturity. The CTDI-CV contains 70 items (10 items per subscale). It simplifies the CCTDI scoring procedure while retaining the same subscale and total scoring points. The scale has shown good reliability in Chinese university L2 populations in previous studies (e.g., Jie & Boon, 2024; Liu & Yao, 2019; Peng et al., 2004). In the present study, the internal consistency reliability of the total score was adequate (Cronbach’s *α* = 0.88).

#### 4.2.4. The Runco Ideational Behavior Scale

Creativity was measured with the Runco Ideational Behavior Scale (RIBS; Runco et al., 2001). The scale has 23 items describing everyday ideational behaviors, rated from 1 (“never”) to 5 (“very often”). The scale was translated from English into Chinese by the authors. The translation was carefully reviewed and revised to ensure that it was appropriate and conveyed the intended meanings of the items for the target population of Chinese university students. The internal consistency of the scale in the current sample was excellent (Cronbach’s *α* = 0.93). To examine the latent structure of the RIBS scale in the current sample, we conducted an exploratory factor analysis (EFA) using principal axis factoring (PAF) with no rotation. The data were suitable for factor analysis (KMO = 0.924, Bartlett’s test of sphericity *χ*^2^ (136) = 1217.448, *p* < 0.001). The one-factor model fit the data well, explaining about 44% of the variance. This aligns with previous validation studies and justifies treating creativity as a composite score. These results provide preliminary evidence for the structural adequacy of the RIBS in the current sample.

### 4.3. Data Analysis

SPSS (v25.0) was used for descriptive statistics and correlation analyses. Hypothesis testing employed the PROCESS macro (v3.2; Model 4/6) with 5000 bootstrap resamples to estimate indirect effects and 95% confidence intervals (CIs). We followed established procedures for mediation analysis (Hayes, 2022; Preacher & Hayes, 2004).

### 4.4. Results

#### 4.4.1. Descriptive Statistics and Correlation Analysis

Participants’ performance in AI self-efficacy (102.096 ± 12.375), AI literacy (65.415 ± 6.822), critical thinking disposition (277.867 ± 23.665), and creativity (55.674 ± 10.626) was evaluated (Table 4). Pearson correlations indicated that AI self-efficacy was positively related to AI literacy (*r* = 0.328, *p* < 0.001), critical thinking disposition (*r* = 0.320, *p* < 0.001), and creativity (*r* = 0.319, *p* < 0.001). AI literacy was positively associated with critical thinking disposition (*r* = 0.349, *p* < 0.001) and creativity (*r* = 0.340, *p* < 0.001). In addition, critical thinking disposition correlated with creativity (*r* = 0.385, *p* < 0.001).

#### 4.4.2. Mediation Analysis

**Figure 2:**
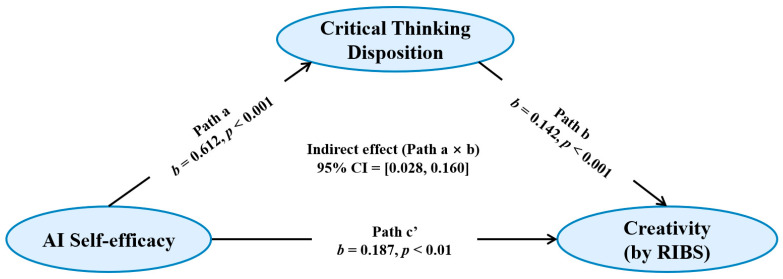
The mediation role of critical thinking disposition in the correlation between AI self-efficacy and creativity (H2).

To test Hypothesis 2 (H2), a simple mediation analysis was performed (Figure 2). The results indicated that AI self-efficacy positively predicted critical thinking disposition (*b* = 0.612, *SE* = 0.157, *β* = 0.320, *p* < 0.001). This model explained 10.2% of the variance in critical thinking disposition (*R*^2^ = 0.102, *f*^2^ = 0.114). The full model, including both AI self-efficacy and critical thinking disposition, explained 19.1% of the variance in creativity (*R*^2^ = 0.191). Within this model, AI self-efficacy positively predicted creativity (*b* = 0.187, *SE* = 0.071, *β* = 0.218, *p* < 0.01), and critical thinking disposition also positively predicted creativity (*b* = 0.142, *SE* = 0.037, *β* = 0.315, *p* < 0.001). Additionally, the bootstrapped indirect effect of AI self-efficacy on creativity through critical thinking disposition was significant (*b* = 0.087, *SE* = 0.034, 95% CI = [0.028, 0.160]; completely standardized indirect effect = 0.101, *SE* = 0.039, 95% CI [0.032, 0.187]). These results suggest that critical thinking disposition mediates the relationship between AI self-efficacy and creativity. Because the serial mediation analysis was conducted using PROCESS Model 6, some coefficients reported in this section correspond to the same structural paths within the same model and therefore appear numerically identical.

**Figure 3:**
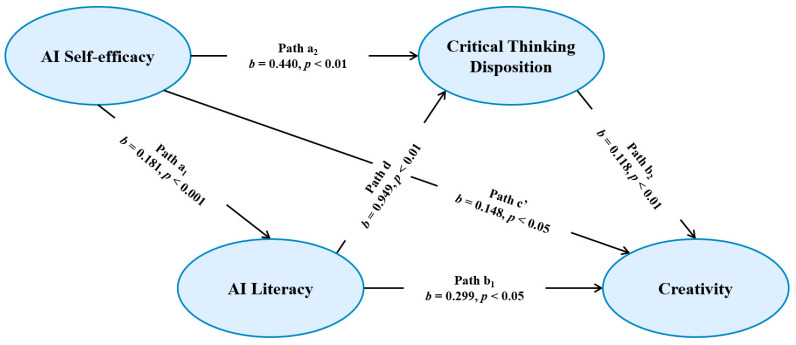
The chain mediation role of AI literacy and critical thinking disposition in the correlation between AI self-efficacy and creativity (H3).

To evaluate Hypothesis 3 (H3), we estimated a serial mediation model in which AI self-efficacy predicts creativity through AI literacy and critical thinking disposition (Figure 3). AI self-efficacy positively predicted AI literacy (*b* = 0.181, *SE* = 0.045, *β* = 0.328, *p* < 0.001). This model explained 10.8% of the variance in AI literacy (*R*^2^ = 0.108, *f*^2^ = 0.121). When predicting critical thinking disposition from AI self-efficacy and AI literacy, the model accounted for 16.9% of the variance in critical thinking disposition (*R*^2^ = 0.169, *f*^2^ = 0.203). In this model, AI self-efficacy positively predicted critical thinking disposition (*b* = 0.440, *SE* = 0.161, *β* = 0.230, *p* < 0.01). AI literacy was also positively associated with critical thinking disposition (*b* = 0.949, *SE* = 0.291, *β* = 0.274, *p* < 0.01). The full model, including AI self-efficacy, AI literacy, and critical thinking disposition, explained 22.1% of the variance in creativity (*R*^2^ = 0.221). Within this model, AI self-efficacy (*b* = 0.148, *SE* = 0.072, *β* = 0.172, *p* < 0.05), AI literacy (*b* = 0.299, *SE* = 0.132, *β* = 0.192, *p* < 0.05), and critical thinking disposition (*b* = 0.118, *SE* = 0.038, *β* = 0.263, *p* < 0.01) predicted creativity. The serial indirect effect (i.e., AI self-efficacy → AI literacy → critical thinking disposition → creativity) was significant (*b* = 0.020, *SE* = 0.013, 95% CI [0.003, 0.050]; completely standardized indirect effect = 0.024, *SE* = 0.014, 95% CI [0.004, 0.056]). It indicates that the two mediators operate sequentially.

The results showed that the total indirect effect and three significant indirect pathways were significant, while the direct effect remained significant (Table 5).

#### 4.4.3. Post Hoc Sensitivity (Power) Analysis

We performed a post hoc sensitivity (power) analysis in G*Power for the multiple regression equation(s) that underlie the PROCESS serial mediation steps, using the omnibus *F* test for each regression equation. For Study 2, the achieved power was 0.999 (*α* = 0.05, *N* = 135, number of tested predictors = 3, and total number of predictors = 4), with a critical *F* of 2.674. These results suggest high sensitivity at the regression-equation level for the observed effects in Study 2.

### 4.5. Validation and Robustness Checks

Several checks were conducted to assess the robustness of the findings from Study 2.

First, given that the focal predictors and mediators were self-reported, Harman’s single-factor test was employed to assess common-method variance. The first unrotated factor accounted for 16.40% of the total variance, which is well below the commonly used 50% benchmark. It suggests that common-method bias is unlikely to account for a substantial portion of the observed associations.

Second, we examined multicollinearity across the regression models. Tolerance values were found to exceed 0.20, and VIF values were found to be below 5.00. The results indicate multicollinearity was not a major concern.

Third, the serial mediation model was re-estimated, with the age of onset of English learning and self-rated English proficiency as covariates in all equations. The overall pattern of direct (*b* = 0.268, *SE* = 0.071, *p* < 0.001) and indirect effects (*b* = 0.128, *SE* = 0.036, 95% CI [0.062, 0.204]) remained unchanged, while the serial indirect effect through AI literacy and critical thinking disposition remained significant (*b* = 0.020, *SE* = 0.012, 95% CI [0.003, 0.049]).

### 4.6. Interim Discussion

Study 2 extended the findings of Study 1 by utilizing a larger participant sample and a different creativity assessment (i.e., the RIBS). The results indicated that both AI literacy and critical thinking disposition were associated with creativity in L2 learners. Simple mediation analysis provided support for the second hypothesis (H2). Moreover, the chain mediation analysis suggested that AI literacy and critical thinking disposition may operate sequentially in the relationship between AI self-efficacy and creativity, providing support for the third hypothesis (H3). These findings suggest that AI self-efficacy is associated with creativity both directly and indirectly through AI literacy and critical thinking disposition. It highlights the role of AI competencies and evaluative tendencies in AI-supported L2 learning contexts.

## 5. General Discussion

Prior studies have established positive associations between AI literacy, critical thinking, and creativity (Dwyer et al., 2025; Naveed et al., 2023; Shaber et al., 2025; M. Zhou & Peng, 2025). However, little empirical work has examined how AI self-efficacy predicts creativity. This issue is particularly significant in L2 learners, who have special multilingual experiences, cultural diversity, and cognitive strengths. To address this gap, the present study used mediation analysis to examine how AI self-efficacy predicts L2 learners’ creativity through AI literacy and critical thinking disposition. Results revealed both direct and indirect associations between AI self-efficacy and creativity. Learners who reported stronger confidence in their ability to use AI tended to demonstrate higher levels of creativity. Beyond this direct association, AI self-efficacy also predicted creativity indirectly through three pathways, i.e., AI literacy and critical thinking disposition, respectively and sequentially.

### 5.1. AI Self-Efficacy and Creativity

The present findings suggest that AI self-efficacy is positively associated with creativity in AI-supported L2 learning contexts. This pattern is consistent with prior research demonstrating that self-efficacy beliefs are related to creative performance across educational and professional settings (Haase et al., 2018; Tao et al., 2025; Tierney & Farmer, 2002). Language learners who feel confident in their ability to use AI tools may be more likely to engage actively in learning tasks, experiment with alternative ideas, and persist when encountering uncertainty. These qualities have been identified as important for creative thinking (Bandura, 1997; Teo, 2019).

From a social cognitive perspective, self-efficacy beliefs shape individuals’ motivation, effort investment, and self-regulatory behavior (Bandura, 1997). Applied to AI-supported L2 learning, AI self-efficacy may influence how learners use human–AI interaction, including whether they explore multiple AI outputs, refine prompts, or reflect on generated information. Such engagement patterns, in theory, are linked to creative idea generation, particularly in L2 learning tasks that require flexibility and originality. However, it should be emphasized that the present findings do not imply that AI self-efficacy directly enhances creativity. Rather, the results indicate predictive associations that are consistent with social cognitive theory.

At the same time, recent studies have cautioned that AI use may also have potential negative implications for creativity, including overreliance on AI output, reduced cognitive effort, or homogenization of ideas (Gerlich, 2025; Zhai et al., 2024). Accordingly, the positive association observed in the present study should be interpreted in relation to how AI is used and regulated in L2 learning contexts. AI self-efficacy may support creativity when it is accompanied by reflective engagement and critical evaluation (i.e., critical thinking), but it may not do so when AI is used uncritically or as a substitute for independent thinking.

### 5.2. The Mediation Role of AI Literacy

The current research found that AI self-efficacy positively predicts L2 learners’ AI literacy. Previous studies have focused on the reverse direction, namely that AI literacy can enhance AI self-efficacy (He et al., 2025; Yuan et al., 2024). However, from a social cognitive theory perspective (Bandura, 1997), self-efficacy is a key determinant of individuals’ willingness to engage with and persist in learning activities. Individuals with higher AI self-efficacy are more likely to actively explore AI tools, invest time in mastering their functions, and persist in overcoming technical difficulties. Such sustained engagement may provide more opportunities for language learning and the gradual development of both technical competence and critical evaluative skills, which are central components of AI literacy. Similar patterns have been observed in the broader digital literacy domain, where higher technological self-efficacy predicts subsequent gains in digital skills through increased usage and exploratory behaviors. Research has also shown that AI literacy and AI self-efficacy are interrelated (Zeng et al., 2025). Taken together, these findings indicate that AI self-efficacy is closely linked to the development of AI literacy.

In addition, our findings revealed that AI literacy is positively associated with creativity. AI literacy is defined as “a set of competencies that enables individuals to evaluate AI technologies; communicate and collaborate effectively with AI; and use AI as a tool online, at home, and in the workplace” (Long & Magerko, 2020). Individuals who possess higher AI literacy are better able to use AI tools for divergent thinking, combine multimodal resources to generate novel ideas, and refine AI output to achieve creative goals. Recent reviews further suggest that tools such as ChatGPT expand learning opportunities and provide personalized support that may facilitate innovative thinking (Lo et al., 2024). Empirical evidence also shows that higher AI literacy is positively associated not only with academic performance, such as writing ability, but also with psychological well-being in AI-supported contexts (Shi et al., 2025).

The observed association between AI literacy and creativity is consistent with recent empirical work. M. Zhou and Peng (2025) found that AI literacy moderated the effect of instructional activities on students’ creativity through enhanced learning engagement. It suggests that higher AI literacy enables more effective and innovative use of AI resources. Similarly, Ji et al. (2025) provided further evidence that AI literacy is positively associated with innovative performance indirectly through psychological and self-regulatory mechanisms in learning tasks. These findings align with the theoretical proposition that literacy in emerging technologies does not merely facilitate functional use but also supports cognitive capacities relevant to creative performance.

Consistent with our first hypothesis (H1), results of the present study demonstrate that AI self-efficacy predicts creativity both directly and indirectly through AI literacy. This pattern is consistent with prior research suggesting that AI competencies and efficacy beliefs may jointly shape creativity outcomes (McGuire et al., 2024; M. Zhou & Peng, 2025). In other words, AI self-efficacy appears to initiate a series of cognitive and behavioral processes, which ultimately contribute to creative output.

### 5.3. The Mediation Role of Critical Thinking Disposition

The present study found that AI self-efficacy positively predicts critical thinking disposition among L2 learners. This finding is consistent with social cognitive theory, which emphasizes the role of efficacy beliefs in shaping individuals’ engagement, self-regulation, and cognitive effort (Bandura, 1997). Learners with higher AI self-efficacy are more likely to approach AI-supported tasks with confidence, to remain engaged when AI outputs require evaluation or revision, and to invest effort in monitoring and regulating their interactions with AI systems. Such engagement tendencies are closely aligned with the dispositional nature of critical thinking as measured in the present study.

Previous research has demonstrated that self-efficacy beliefs in academic and technological domains are associated with stronger evaluative engagement, critical thinking, or credibility judgment (Chang et al., 2022; Y. P. Wang et al., 2022; Anttonen et al., 2024). These findings provide indirect support for a possible connection between self-efficacy and critical thinking disposition. In AI-supported learning environments, dispositional tendencies are particularly important because AI outputs often present information with high surface fluency but variable accuracy. Language learners must therefore decide whether to accept, modify, or reject AI suggestions. Critical thinking disposition, rather than critical thinking ability per se, captures learners’ habitual inclination to engage in such evaluative processes.

In addition, critical thinking disposition was found to be positively associated with creativity. This finding is consistent with prior research suggesting that creativity and critical thinking are complementary rather than opposing processes (Dwyer et al., 2025). While creativity emphasizes idea generation and originality, critical thinking disposition supports the evaluation, refinement, and contextual appropriateness of those ideas. In AI-supported L2 learning, learners who are inclined to question information, consider alternatives, and justify decisions may be better able to transform AI responses into creative and meaningful outputs.

Consistent with the second hypothesis (H2), critical thinking disposition served as a mediator between AI self-efficacy and creativity. This pattern suggests that language learners who feel confident in their ability to use AI tools are more likely to engage in evaluative and reflective thinking (i.e., critical thinking disposition), which in turn is associated with higher levels of creativity. Importantly, these findings should not be interpreted as evidence that AI self-efficacy directly enhances critical thinking ability or creativity. Rather, the results indicate predictive associations that are consistent with social cognitive accounts but do not establish causal directionality.

### 5.4. The Chain Mediation Role of AI Literacy and Critical Thinking Disposition

The present study further examined whether AI literacy and critical thinking disposition mediate sequentially the association between AI self-efficacy and creativity. The chain mediation analysis provided evidence that AI self-efficacy was predictively related to AI literacy, which in turn was associated with critical thinking disposition, and that these two variables were associated with creativity. This serial indirect effect was significant, while the direct path from AI self-efficacy to creativity remained significant. This finding supports the third hypothesis (H3).

Importantly, this chain pattern does not merely duplicate the independent mediation effects reported in Section 5.2 and Section 5.3. Instead, it suggests that AI literacy and critical thinking disposition may function as linked but distinguishable stages within AI-supported L2 learning processes. AI literacy reflects learners’ knowledge and evaluative resources for engaging with AI tools (Long & Magerko, 2020), whereas critical thinking disposition captures learners’ habitual tendency to apply those resources during language learning activities. Therefore, the chain mediation highlights how competence and dispositional factors may be coordinated rather than operating in isolation.

From a theoretical perspective, this sequential pattern is consistent with social cognitive theory. According to the theory, efficacy beliefs are linked to engagement, engagement is associated with competence development, and competence supports reflective regulation of thinking and action (Bandura, 1997; Schunk & DiBenedetto, 2020; Zimmerman, 2000). Rather than implying a strict causal progression, the present findings are interpreted as theory-driven predictive associations that help clarify how multiple constructs may jointly relate to creativity in AI-supported L2 learning contexts. In this sense, creativity appears to be linked not only to language learners’ confidence in using AI but also to the coordinated contribution of AI literacy and critical thinking disposition.

### 5.5. Pedagogical Implications for L2 Learning

From a pedagogical perspective, language educators are well placed to design classroom environments that encourage students to engage with AI tools in an exploratory and reflective way. Rather than presenting AI as a source of answers, instructors can set tasks that require language students to be critical and evaluate AI responses, compare multiple outputs, and justify their revisions based on explicit criteria such as relevance, evidence, and logical coherence. Such tasks have been shown to enhance AI literacy by familiarizing individuals with various AI functions, prompt engineering techniques, and methods for assessing reliability (e.g., identifying unsupported claims or potential bias). At the same time, they encourage critical thinking disposition through sustained questioning and reasoning.

Educators may also implement collaborative projects in which language students work together to use AI to conceptualize, develop, and refine creative works (e.g., narratives, debates, and multimedia presentations). For example, leaner groups can be asked to document their AI prompts, compare alternative AI outputs, and justify why certain ideas are adopted, revised, or rejected. These projects could be accompanied by guided reflection on how AI contributed to idea generation and how human judgment ultimately shaped the final product. Requiring students to write an initial draft before using AI, then revise iteratively with AI support, may also reduce uncritical reliance on AI tools.

Integrating AI use into authentic problem-solving scenarios can turn the classroom into a useful practice space for developing AI literacy and critical thinking disposition. Assessment strategies may therefore benefit from emphasizing process, such as revision logs, evaluation notes, and reflective journals, rather than focusing on final products. This integrated approach can encourage L2 learners’ creativity in a way that is both intentional and transferable to real-world scenarios where AI-assisted communication and decision-making are increasingly common.

### 5.6. Limitations and Future Directions

Several limitations of the present study should be acknowledged.

First, the cross-sectional design limits causal inference regarding the relationships among AI self-efficacy, AI literacy, critical thinking disposition, and creativity. Although the mediation models were theoretically grounded and tested using bootstrapped indirect effects, the findings should be interpreted as predictive associations rather than evidence of causal mechanisms. Reciprocal relationships are also possible in theory. Future research using longitudinal or experimental designs is needed to investigate temporal ordering and bidirectional dynamics among these constructs.

Second, creativity was measured using different methods across the two substudies, with a divergent thinking task in Study 1 and a self-report measure of everyday ideational behavior in Study 2. While convergence across these measures provides preliminary cross-operational evidence, they capture distinct facets of creativity and may differ in task demands and susceptibility to response bias. In addition, some indirect effects, particularly the serial mediation pathway, were relatively small in magnitude. Future studies with larger samples and higher statistical power are needed to obtain more precise estimates and to test intervention effects.

Third, all key variables were assessed using self-report measures, which may introduce common method bias and reflect perceived rather than objective competence. Although statistical checks suggested that common method variance was unlikely to account for the findings, future research should incorporate behavioral, performance-based, or trace measures of AI use, AI literacy, and critical thinking disposition.

Finally, the sample consisted of Chinese university students learning English, which may limit generalizability. Future replication studies across different cultural, disciplinary, and instructional contexts are therefore necessary to assess the robustness and external validity of the proposed model.

## 6. Conclusions

The present study addressed critical gaps in research on how AI and creativity are associated by focusing on L2 learners. This population is significant for investigating higher-order thinking skills due to their multilingual experiences, multicultural exposure, and cognitive advantages. While earlier studies have established a correlation between AI literacy and critical thinking disposition with creativity, the present study is among the first to examine how AI self-efficacy predicts creativity through these variables in AI-supported L2 learning contexts.

Findings from two interrelated substudies demonstrated that AI self-efficacy positively predicted creativity both directly and indirectly through the mediating effects of AI literacy and critical thinking disposition. In addition, a sequential pathway was also identified. It shows that AI self-efficacy was associated with higher levels of AI literacy, which in turn were associated with stronger critical thinking disposition. Critical thinking disposition was further linked to creativity. While these results are consistent with a social cognitive perspective, they should be interpreted as theory-consistent predictive associations rather than evidence of causal mechanisms.

Overall, the current study contributes to the growing literature on AI-supported L2 learning by highlighting the role of efficacy beliefs and evaluative competencies in creativity development. It brings together AI self-efficacy, AI literacy, and critical thinking in a unified framework and gives us a clear picture of how language learners should think about and use AI tools. The findings show that simply providing AI access is not enough. Educators also need to help language learners build confidence, think critically about AI outputs, and use these tools reflectively.

## Figures and Tables

**Table 1:** Demographic Information of Study 1.

Demographic Characteristic	*M* ± *SD*
1 Age	19.310 ± 0.973
2 Age of Onset of English	8.236 ± 1.756
3 Self-reported English Proficiency	4.125 ± 0.580

**Table 2:** Descriptive Statistics and Pearson Correlations for the Variables in Study 1.

Variables	*M* ± *SD*	1	2	3
1 AI Self-efficacy	101.653 ± 12.907	1	0.371 **	0.400 ***
2 AI Literacy	65.125 ± 7.403	0.371 **	1	0.367 **
3 Creativity (AUT)	5.770 ± 1.273	0.400 ***	0.367 **	1

** *p* < 0.01, *** *p* < 0.001.

**Table 3:** Demographic Information of Study 2.

Demographic Characteristic	*M* ± *SD*
1 Age	19.470 ± 1.164
2 Age of Onset of English	8.100 ± 1.912
3 Self-reported English Proficiency	4.160 ± 0.660

**Table 4:** Descriptive Statistics and Pearson Correlations for the Variables in Study 2.

Variables	*M* ± *SD*	1	2	3	4
1 AI Self-efficacy	102.096 ± 12.375	1	0.328 ***	0.320 ***	0.319 ***
2 AI Literacy	65.415 ± 6.822	0.328 ***	1	0.349 ***	0.340 ***
3 Critical Thinking Disposition	277.867 ± 23.665	0.320 ***	0.349 ***	1	0.385 ***
4 Creativity	55.674 ± 10.626	0.319 ***	0.340 ***	0.385 ***	1

*** *p* < 0.001.

**Table 5:** Direct and indirect effects of AI self-efficacy on creativity.

	*b*	*SE*	LLCI	ULCI
**Total Effect**	0.274	0.071	0.134	0.414
**Direct Effect**				
AI self-efficacy → Creativity	0.148	0.072	0.005	0.290
**Indirect Effect**				
AI self-efficacy → AI literacy → creativity	0.054	0.027	0.006	0.112
AI self-efficacy → critical thinking disposition → creativity	0.052	0.029	0.005	0.116
AI self-efficacy → AI literacy → critical thinking disposition → creativity	0.020	0.013	0.003	0.050
**Total Indirect effect**	0.126	0.037	0.057	0.204

## Data Availability

The original data are not available online due to restrictions/confidentiality in the authors’ institutions/programs. Researchers can apply to get access to the data from the authors’ institutional ethics committees (Email: liyadan@snnu.edu.cn).
